# Cordycepin mediates pyroptosis in HCC through the upregulation of TXNIP and synergizes with anti–PD-L1 immunotherapy

**DOI:** 10.1097/HC9.0000000000000633

**Published:** 2025-02-26

**Authors:** Bu-Gang Liang, Yi-Min Zheng, Hong-Ye Shen, Guo-Huan Yang, Wen-Xin Xu, Chang-Jun Tan, Ai-Wu Ke, Wen-Zheng Qin

**Affiliations:** 1 Department of Liver Surgery and Transplantation, Liver Cancer Institute and Zhongshan Hospital, Fudan University, Shanghai, People’s Republic of China; 2 Department of Liver Cancer Institute, Zhongshan Hospital, Fudan University, Key Laboratory of Carcinogenesis and Cancer Invasion (Fudan University), Ministry of Education, Shanghai, PR China; 3 Department of Academic Affairs Centre, Shanghai Nanyang Model High School, Shanghai, People’s Republic of China; 4 Department of Endoscopy Center, Endoscopy Research Institute, Zhongshan Hospital, Fudan University, Shanghai Collaborative Innovation Center of Endoscopy, Shanghai, China

**Keywords:** anti–PD-L1 immunotherapy, cordycepin, HCC, pyroptosis, TXNIP

## Abstract

**Background:**

Immune checkpoint inhibitors are effective treatments for HCC; however, their therapeutic efficacy is often limited by the development of drug resistance. Therefore, investigating new combination therapeutics involving immune checkpoint inhibitors is critical to improving patient prognosis. In this study, we investigated the therapeutic effect of cordycepin (COR) in HCC and its synergistic effect with anti–programmed cell death ligand 1 (anti–PD-L1) immunotherapy.

**Methods:**

We selected 2 HCC cell lines to investigate the effects of COR on HCC growth using in vivo and in vitro experiments. We performed RNA sequencing of the MHCC97H cell line treated with or without COR to understand the underlying mechanism and identify the key regulatory genes. Through in vivo and in vitro experiments on gene knockdown cells, we identified thioredoxin-interacting protein as a key molecule involved in the role of COR. Next, we used mouse subcutaneous and orthotopic tumor models to evaluate the therapeutic effects of COR, atezolizumab (a programmed death-ligand 1 [PD-L1] inhibitor), or their combination. Multiple immunofluorescence staining revealed that the combination of atezolizumab and COR therapy greatly increased the number of tumor-infiltrating CD8^+^ T cells and PD-L1 expression in HCC compared to monotherapy.

**Results:**

Our study revealed that COR significantly inhibited HCC growth both in vitro and in vivo. Mechanistically, we showed that COR induces endoplasmic reticulum stress, which upregulates thioredoxin-interacting protein expression and leads to HCC cell pyroptosis. In addition, the combination treatment with COR and PD-L1 inhibitors profoundly inhibited HCC.

**Conclusions:**

Overall, our study successfully established a combined therapeutic strategy using COR and PD-L1 inhibitors. This strategy has significant synergistic effects on cancer cells, highlighting its importance in cancer therapy.

## INTRODUCTION

HCC accounts for 75%–85% of primary liver cancers and is the most prevalent type of cancer.^1^ It is the fifth most common cancer and the second leading cause of cancer-related mortality worldwide.^2^ Current treatments for liver cancer are mainly divided into surgical and systemic pharmacological therapies.^3^ However, owing to strict patient candidacy requirements, challenges in obtaining suitable liver donors, and high rates of postoperative recurrence, the use of surgical options, such as hepatic resection and liver transplantation, is limited.^4^ Systemic pharmacological therapies include methods such as chemotherapy, immunotherapy, and targeted therapy. Nevertheless, these treatments are plagued by significant side effects and the propensity for drug resistance.^5^ Thus, exploring novel therapeutic agents offers significant potential for improving the overall management of patients with liver cancer.

Compared to chemically synthesized drugs, drugs from natural sources are considered safer. Cordycepin (COR), the active extract of *Cordyceps militaris*, was first discovered by German scientists.^6^ Since then, COR has sparked widespread research interest, particularly regarding its potential role in oncological therapeutics.^7^ Some studies demonstrated that COR inhibited the proliferation of pancreatic cancer cells by arresting the cell cycle and blocking the synthesis of DNA and RNA.^7^,^8^ COR can also induce apoptosis in breast cancer cells.^9^ In addition to exerting direct antitumor effects, COR may positively reshape the tumor microenvironment and enhance immune cell activity and inflammatory responses.^10^ The potential to enhance the effects of chemotherapy in osteosarcoma also increases the likelihood of its use in combination with other drugs.^11^ Although significant progress has been made in the use of COR in oncological research, the possible effects of COR on HCC and the specific mechanisms involved have not been elucidated.

Cellular pyroptosis is a novel type of programmed cell death mediated by the formation of the nucleotide-binding oligomerization structural domain (NOD)-like receptor protein 3 (NLRP3) inflammasome complex. NLRP3 activates and cleaves pro-Caspase-1 to form active Caspase-1, which cleaves gasdermin D (GSDMD) proteins to form active N-terminus and C-terminus.^12^,^13^ The N-terminus contributes to the perforation of the cell membrane, induces cell death, and increases the release of lactate dehydrogenase (LDH) and other cytokines.^12^ Endoplasmic reticulum stress is a phenomenon in cell biology that refers to the abnormal endoplasmic reticulum function, resulting in the accumulation of unfolded or misfolded proteins in the lumen of the endoplasmic reticulum, which triggers a series of cellular stress responses.^14^ Thioredoxin-interacting protein (TXNIP) is a key mediator linking the cellular stress response to the inflammatory response.^15^ TXNIP can be translocated from the nucleus to the mitochondria under conditions of endoplasmic reticulum stress and can activate NLRP3 to mediate inflammatory responses.^16^ Here, we demonstrated that COR mediates pyroptosis in HCC through the upregulation of TXNIP.

Cancer cells can create an immunosuppressive microenvironment that promotes their survival. Programmed death-ligand 1 (PD-L1) is a cell surface protein that plays a critical role in immune regulation by binding to the PD-1 receptor on T cells, leading to immune suppression and inhibition of T-cell–mediated cytotoxicity.^17^ It is often overexpressed in various cancers and contributes to immune evasion. Thus, improving the tumor immune microenvironment has become a key research focus. Recently, clinical trials with immune checkpoint inhibitors, such as anti–PD-L1 and anti–PD-1 antibodies, have shown unprecedented responses in some patients with HCC.^17^,^18^ However, only a limited number of patients respond to immunotherapy, and no biomarkers are available to predict HCC response.^17^,^19^ Immunotherapy failure can be attributed to various factors, including abnormal tumor cell metabolism and limited infiltration of effector T cells. There is an urgent clinical need to improve the efficacy of immune checkpoint inhibitor immunotherapy and identify new combination treatment approaches for patients with HCC.

In this study, we explored the anticancer effects of COR on HCC cells and demonstrated for the first time that COR can mediate tumor cell pyroptosis through the upregulation of TXNIP under ERS. These novel findings provide important clues for an in-depth understanding of the antitumor mechanism of COR. In addition, we confirmed that COR, when used in combination with PD-L1 inhibitors, exerts synergistic cytotoxic effects on tumors. These findings may provide new insights into future combination immunotherapy strategies.

## METHODS

### In vivo tumor experiments

All animal experiments were approved by the Animal Ethics Committee of Zhongshan Hospital, Fudan University, China. Six-week-old male BALB/c-nu/nu or C57BL/6 mice, bred and housed in specific pathogen-free animal facilities at Zhongshan Hospital, were used for the experiments. For xenograft experiments in vivo, stable shTXNIP-expressing or negative control MHCC97H cells (5 × 10^6^ cells resuspended in 100 μL of PBS; the construction methods are presented in the Supplemental Information, http://links.lww.com/HC9/B879) were subcutaneously implanted into the right flank of BALB/c-nu/nu mice (6 mice per group). Tumor size was measured every 3 days using calipers, and tumor volume was calculated as *V* = (length × width^2^​​​​​​)/2. Treatment with COR (150 mg/kg through oral gavage, once every 3 days) commenced on the sixth day and continued for 15 days. Mice treated with vehicle (2% DMSO, 40% PEG400, or 5% Tween-80 in normal saline) were used as controls. Other drugs, including polyphyllin VI (PPVI) (administered at 10 mg/kg/d through i.p. injection) and atezolizumab (administered at 10 mg/kg through i.p. injection, once every 3 days), were prepared according to the manufacturer’s protocol. Tumors were harvested at the end of the experiment and fixed in 10% neutral-buffered formalin. For immunotherapy combination experiments, Hepa1-6 cells (4 × 10^6^ cells resuspended in 100 μL of PBS) were subcutaneously injected into C57BL/6 mice (male, 6–8 wk old) to induce subcutaneous tumors or a suspension of 20 μL of Hepa1-6 cells mixed with Matrigel was injected into the liver using a microinjector to induce the orthotopic tumor model. Treatment with different drugs commenced on the sixth day and was continued for 15 days. At the conclusion of the experiment, tumors or livers were harvested for further analysis and subsequent research. The animals were euthanized according to the norms of the American Veterinary Medical Association (AVMA) to use them scientifically and humanely and to avoid suffering or pain. We first anesthetized the mice with isoflurane to minimize suffering. The induction concentration of isoflurane was 2%–5% and the maintenance concentration was 1%–3%. The animals were considered fully anesthetized when the rollover reflex and muscle tone were lost. Next, the mice were euthanized by injecting sodium pentobarbital. The death of mice was confirmed by assessing the loss of heartbeat, respiration, and reflexes. If necessary, secondary methods, such as cervical dislocation, were used to ensure complete euthanasia. Permission for animal experiments was obtained from the Animal Care and Use Committee of Fudan University.

### Extreme limiting dilution analysis

Extreme limiting dilution analysis was used to assess the effect of COR on the tumor-initiating cells. For the extreme limiting dilution analysis experiments, subcutaneous tumors were harvested from nude mice treated with or without COR and dissociated into single-cell suspensions through enzymatic digestion. Tumor cells were counted using a hemocytometer and viability was confirmed by trypan blue exclusion. The cells were then diluted to the following concentrations: 500, 250, 100, and 50 cells/µL, and 100 µL of each cell suspension (containing 50,000, 25,000, 10,000, or 5000 cells) was injected subcutaneously into nude mice, with 12 mice per group (n = 12 per dilution). The mice were monitored regularly for tumor formation over a period of 4 weeks, and tumor formation was recorded as either positive or negative for each mouse. According to institutional animal care guidelines, mice were euthanized immediately upon the detection of tumor formation. The data were calculated using the extreme limiting dilution analysis method on the website (https://bioinf.wehi.edu.au/software/elda/index.html) to estimate the frequency of tumor-initiating cells and *p* values.

### Observation of cell morphology

The cells were cultured for 24 hours in a regular culture medium or a medium supplemented with the drug of interest. Subsequently, changes in cell morphology were observed using an inverted microscope (Olympus).

### LDH release assays

LDH release in the cell culture supernatant was analyzed using the CytoTox 96 Non-Radioactive Cytotoxicity Assay Kit (Promega, Catalog number G1780) according to the manufacturer’s protocol.

### Tissue microarray and immunohistochemistry

Tissue microarrays comprising 270 paired samples of HCC and matched adjacent noncancerous tissues were collected from the Department of Hepatocellular Carcinoma, Zhongshan Hospital, Fudan University, Shanghai, China. Samples were collected between 2020 and 2023. All samples were immediately flash-frozen in liquid nitrogen after surgical resection and embedded in paraffin wax for tissue microarray construction. Informed consent for the use of samples was obtained from all participants involved in the study. All procedures involving human subjects were approved by the Ethics Committee of Fudan University. For immunohistochemistry, the tissue sections were deparaffinized using a series of graded ethanol solutions. After incubation with 0.3% hydrogen peroxide for ~20 minutes, antigen retrieval was performed at a near 100 °C temperature for 30 minutes with sodium citrate solution and blocked with 5% bovine serum albumin for 60 minutes. Then, the slides were incubated with anti-TXNIP (1:200, Abcam, EPR14774) overnight at 4 °C and with HRP-conjugated secondary antibodies at 37 °C for 1 hour. The sections were then incubated with diaminobenzidine (ZSGB-Bio) for color development, followed by hematoxylin staining for nuclear counterstaining. Images were captured using the Case Viewer software (3DHISTECH) and a standard microscope (Olympus). Image-Pro Plus software (version 6.0) was used to quantify expression levels by calculating the average density value of each image.

### Mitochondrial membrane potential and mitochondrial superoxide detection

A JC-1 MitoMP detection kit (MT09, Dojindo) was used to assess changes in mitochondrial membrane potential, while the mtSOX Deep Red fluorescent probe was used to detect mitochondrial superoxide (MT14, Dojindo) according to the supplier’s protocols. These tools enable the evaluation of alterations in mitochondrial membrane potential and the generation of reactive oxygen species.

### Analysis of public databases

RNA sequencing data from the Cancer Genome Atlas Liver Hepatocellular Carcinoma (TCGA-LIHC) cohort were downloaded from a public data repository. Before further analysis, the transcriptome data were normalized using the “limma” package in the R software. Single-sample gene set enrichment analysis was subsequently performed using the “gsva” package in R software to assess immune cell infiltration in tumors and activity enrichment scores for each sample. In addition, the absolute proportions of infiltration of various immune cell types were calculated using the CIBERSORT algorithm by estimating relative subsets of RNA transcripts. Correlation analysis of the 2 genes was performed using the TIMER2.0 database.

### Statistical analysis

An unpaired Student *t* test was used to calculate 2-tailed *p* values and assess the statistical significance of differences between the 2 groups. For comparisons among multiple groups, 1-way ANOVA was performed, followed by Tukey’s post hoc test. The experimental results are presented as mean ± standard deviation. Tumor growth among the different groups was analyzed using a 2-way ANOVA. Kaplan-Meier curves were constructed for survival analysis, which was assessed using the log-rank test. Two-sided Cox analysis was performed for both univariate and multivariate tests. The *p* values shown in the figures are as follows: ns, not significant, *p* > 0.05; *p < 0.05; **p < 0.01; ***p < 0.001; and ****p < 0.0001. Statistical analyses were performed using GraphPad Prism or R software.

All research was conducted in accordance with the Declaration of Helsinki and the Declaration of Istanbul, and all research was approved by the Zhongshan Hospital Research Ethics Committee. Additional experimental information is presented in the Supplementary Information section, http://links.lww.com/HC9/B879.

## RESULTS

### COR inhibits HCC both in vitro and in vivo

**FIGURE 1:**
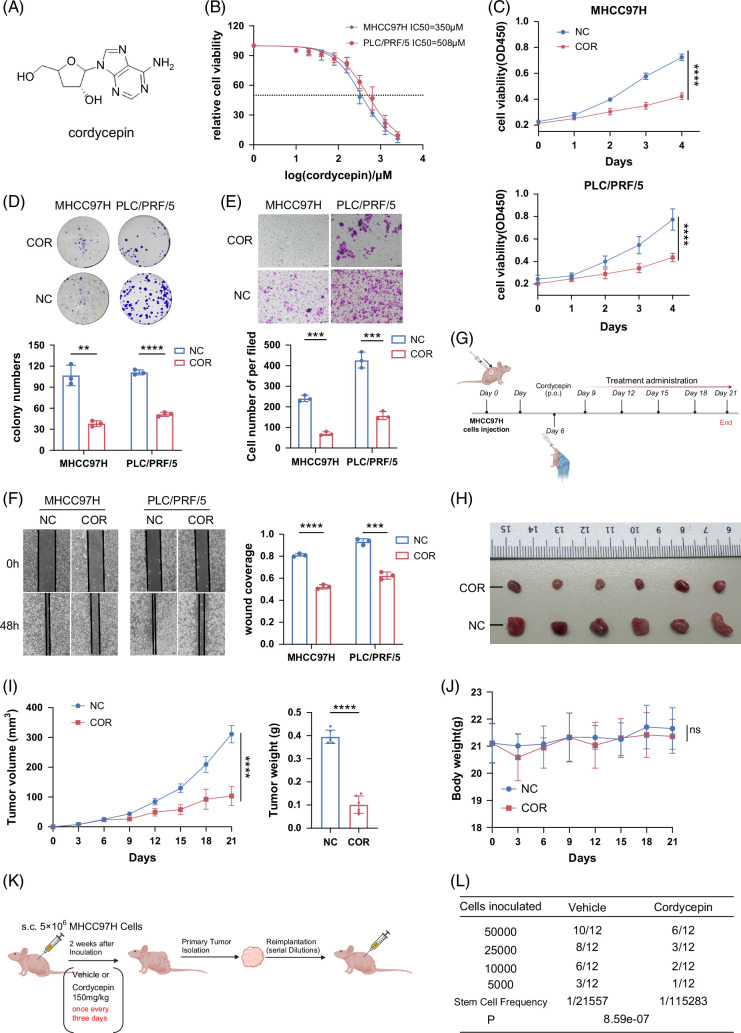
Cordycepin inhibits HCC both in vitro and in vivo. (A) The chemical formula of COR. (B) Determination of the IC50 of COR in 2 cell lines: MHCC97H and PLC/PRF/5. (C) Cell viability in MHCC97H cells under treatment with COR at a concentration of 350 µM and in PLC/PRF/5 cells at 500 µM. (D) Number of colonies formed by PLC/PRF/5 and MHCC97H cells treated with or without COR. (E) Effect of COR on the invasion of PLC/PRF/5 and MHCC97H cells. The concentrations of COR were 50 µM for MHCC97H cells and 100 µM for PLC/PRF/5 cells. (F) Effect of COR on the migration of PLC/PRF/5 and MHCC97H cells. (G) COR treatment strategy for the inhibition of HCC growth in MHCC97H xenografts (n = 6 mice per group). Six days after MHCC97H cell inoculation, COR was administered through the intragastric route at a concentration of 150 mg/kg once every 3 days for a total of 5 times. The mice were sacrificed on day 21. (H–J) Tumor images (H), tumor growth curves, and tumor weights (I) of MHCC97H subcutaneous xenografts treated with or without COR (n = 6 per group). Mouse body weights were shown in (J). (K) Strategy for evaluating the effect of COR on the tumor initiation capacity. (L) COR treatment decreases the TIC frequency in vivo. The frequency of tumorigenic cells and probability estimates were calculated using ELDA software. “P” indicates a statistically significant difference in the TIC frequency between the vehicle and COR modes. The figures in (G) and (J) were created with BioRender.com. The above data are presented as the means ± SDs of 3 independent experiments. *p* values were denoted as follows: ns, not significant, *p* > 0.05; **p < 0.01; ***p < 0.001; and ****p < 0.0001. Abbreviations: COR, cordycepin; ELDA, extreme limiting dilution analysis; TIC, tumor-initiating cell.

We elucidated the structural formula of COR based on previously published findings^20^ and visually represented its chemical formula (Figure 1A). We selected 2 prevalent HCC cell lines, MHCC97H and PLC/PRF/5, for our experimental studies to assess the cytotoxicity of COR on HCC cells. We determined the 50% inhibitory concentration (IC50) of COR in these cell lines (Figure 1B). Based on the IC50 results, we selected different concentrations of COR for cell viability assay and migration assay of MHCC97H cells and PLC/PRF/5 cells. Using CCK8 and colony formation assays (Figures 1C, D), we determined that COR substantially inhibited HCC cell proliferation in vitro. In addition, compared to the negative control, COR markedly inhibited the migration and invasion of HCC cells (Figures 1E, F). Motivated by these promising results in vitro, we established a subcutaneous tumor model in nude mice using MHCC97H cells and administered COR at a concentration of 150 mg/kg (Figure 1G). Our results showed COR strongly suppressed HCC growth in vivo (Figures 1H, I). We also observed no significant alterations in the body weights of mice treated with or without COR (Figure 1J), indicating the safety profile of COR alongside its potent tumor-suppressive effects. Furthermore, our investigations revealed that COR substantially reduced the tumor-initiating cell frequency by nearly 5-fold (1/tumor-initiating cell from ~21,557 cells to ~115,283 cells) (Figures 1K, L), suggesting its potential to suppress HCC stemness. Taken together, our findings underscore the potent antitumor effect of COR against HCC.

### COR more strongly inhibits the growth of HCC cells than its analogs

Since COR has been identified as 3′-deoxyadenosine, we investigated whether other analogs of COR have similar effects on HCC cells. We selected 3 COR analogs, adenosine, 2′-deoxyadenosine, and 2′,3′-dideoxyadenosine (ddAdo), to compare their effects on HCC cells to that of COR (Supplemental Figure S1A, http://links.lww.com/HC9/B879). By determining the IC50 values of these analogs in HCC cell lines, we found that adenosine and 2′-deoxyadenosine had minimal inhibitory effects on HCC cells (IC50 > 5 mM, Supplemental Figure S1B, http://links.lww.com/HC9/B879), whereas ddAdo exhibited some inhibitory effect. When comparing ddAdo to COR, we observed that COR more effectively inhibited tumor cell migration and proliferation under the same conditions (Supplemental Figures S1C–H, http://links.lww.com/HC9/B879). These results indicate that compared with the other analogs, 3′-deoxyadenosine has a stronger antitumor effect. COR may be more effective in disrupting tumor cell proliferation or survival mechanisms, or in activating antitumor pathways, highlighting its potential application in HCC treatment.

### COR can induce endoplasmic reticulum stress and pyroptosis

**FIGURE 2:**
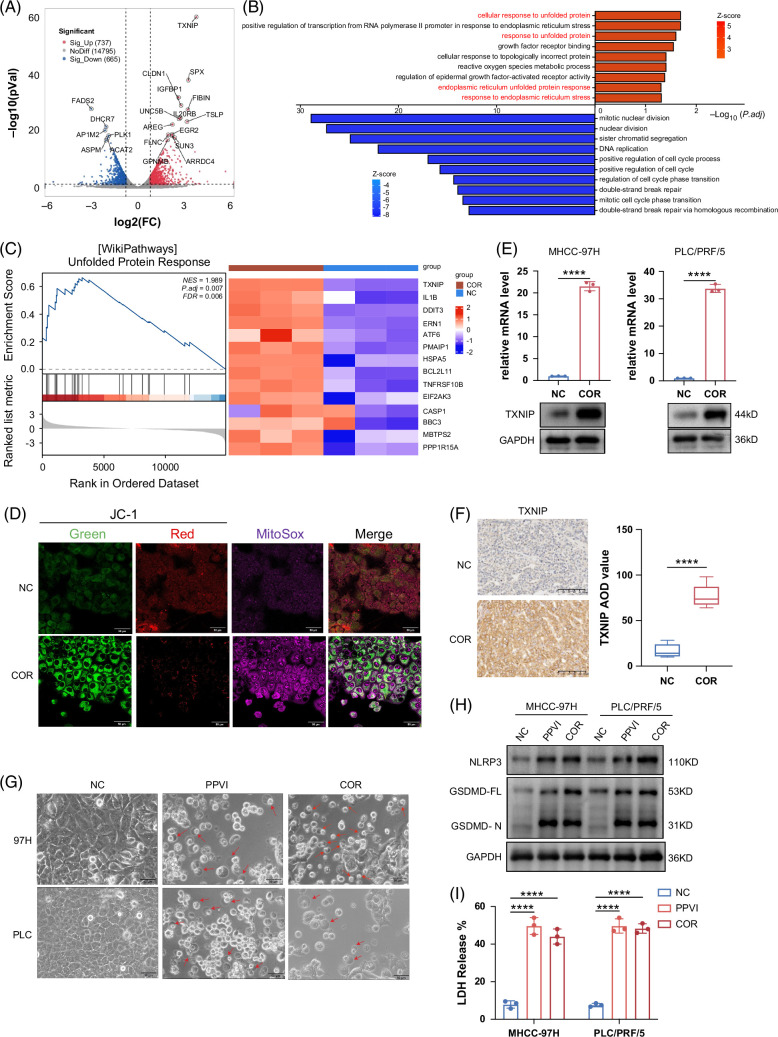
COR can induce ERS and pyroptosis. (A) Volcano map of differentially expressed genes. TXNIP was upregulated in the COR group. (B) Joint GO/KEGG FC analyses of differentially expressed genes and labeling of the 10 pathways with significant differences. (C) Unfolded protein response pathway in the negative control and COR groups identified by GSEA (left panel). A heatmap of differentially expressed genes in the corresponding pathways between the negative control and COR treatment groups (right panel). (D) JC-1 MitoMP was used to assess changes in the mitochondrial membrane potential, and the mtSOX Deep Red fluorescent probe was used to detect mitochondrial superoxide levels. PLC/PRF/5 cells were used in this study. When mitochondria are depolarized, the intensity of green fluorescence increases and the intensity of red fluorescence decreases. The purple fluorescence intensifies when mitochondrial superoxide is detected. (E) qPCR and Western blot results showing differences in TXNIP expression between the negative control and COR treatment groups. (F) Immunohistochemical staining of TXNIP in previous subcutaneous tumor sections from the negative control and COR treatment groups. The AOD value was calculated using ImageJ. (G–I) MHCC97H and PLC/PRF/5 cell lines were pretreated with vehicle, PPVI (20 μM), or COR (350 µM for MHCC97H and 500 µM for PLC/PRF/5). Cell pyroptosis was detected by observing the morphology (G, red arrows indicate pyroptotic cells), GSDMD cleavage (H), and LDH release (I). The above data are presented as the means ± SDs of 3 independent experiments. *p* values were denoted as follows: ns, not significant, *p* > 0.05 and ****p < 0.0001. Abbreviations: COR, cordycepin; ERS, endoplasmic reticulum stress; GSEA, gene set enrichment analysis; PPVI, polyphyllin VI; TXNIP, thioredoxin-interacting protein.

We cultured MHCC97H cells in a normal medium or COR-containing medium for 2 days and then performed RNA sequencing to elucidate the mechanism underlying the inhibition of tumor growth by COR (sequencing data are available in the Supplemental Table, http://links.lww.com/HC9/B880). We plotted a volcano plot to visualize the differentially expressed genes, highlighting the top 10 genes with the most significant differences in upregulation and downregulation (Figure 2A). The pronounced upregulation of TXNIP is especially noteworthy, indicating its potential value in our study. We subsequently performed joint GO/KEGG FC analyses of the differentially expressed genes. The 10 pathways with significant differences in upregulation and downregulation are displayed (Figure 2B). The results showed that upregulated pathways were associated with endoplasmic reticulum stress (ERS) or unfolded protein response. Endoplasmic reticulum stress is a cellular phenomenon characterized by the accumulation of unfolded or misfolded proteins in the endoplasmic reticulum lumen that triggers a series of cellular stress responses.^14^ Gene set enrichment analysis also revealed that the gene signatures of the unfolded protein response were significantly enriched in the COR group (Figure 2C). TXNIP also plays an important role in this pathway (Figure 2C). ERS and activation of the unfolded protein response can lead to impaired calcium and redox homeostasis, inducing oxidative stress through protein overload, thereby affecting important mitochondrial functions.^21^ Therefore, we monitored ERS by detecting changes in mitochondrial membrane potential and reactive oxygen species production. Compared to the control group, PLC/PRF/5 cells treated with COR presented more pronounced alterations in the mitochondrial membrane potential and increased reactive oxygen species generation, indicating that COR could induce endoplasmic reticulum stress (Figure 2D). Subsequently, we confirmed that TXNIP expression was notably elevated in the COR-treated group (Figure 2E). Immunohistochemistry staining of resected tissues from previous xenograft tumors revealed high TXNIP expression in the COR group (Figure 2F). Previous studies have shown that under conditions of ERS, TXNIP activates the formation of nucleotide-binding oligomerization domain (NOD)-like receptor protein 3 (NLRP3) inflammasome complexes, subsequently inducing cellular pyroptosis.^15^,^22^ We investigated whether COR could induce pyroptosis in HCC cells. Morphological changes were observed in HCC cells by using the pyroptosis inducer PPVI as a positive control.^23^ Microscopy images showed that cells in both COR and PPVI groups exhibited morphological characteristics of pyroptosis (Figure 2G). Moreover, the COR-treated group exhibited hallmark characteristics of pyroptosis, including the synthesis of NLRP3 vesicles, cleavage of gasdermin D (GSDMD), and release of LDH (Figures 2H, I). These data suggest that COR exerts tumor-suppressive effects by inducing pyroptosis. We also compared the ability of ddAdo and COR to induce TXNIP expression and activate pyroptosis, respectively. Although ddAdo slightly upregulated TXNIP expression (Supplemental Figure S2A, http://links.lww.com/HC9/B879), HCC cells treated with ddAdo did not show significant characteristics of pyroptosis (Supplemental Figures S2B–D, http://links.lww.com/HC9/B879). These findings suggest that COR possesses a unique structure that effectively activates cell pyroptosis.

### COR induces pyroptosis in HCC cells through the upregulation of TXNIP

**FIGURE 3:**
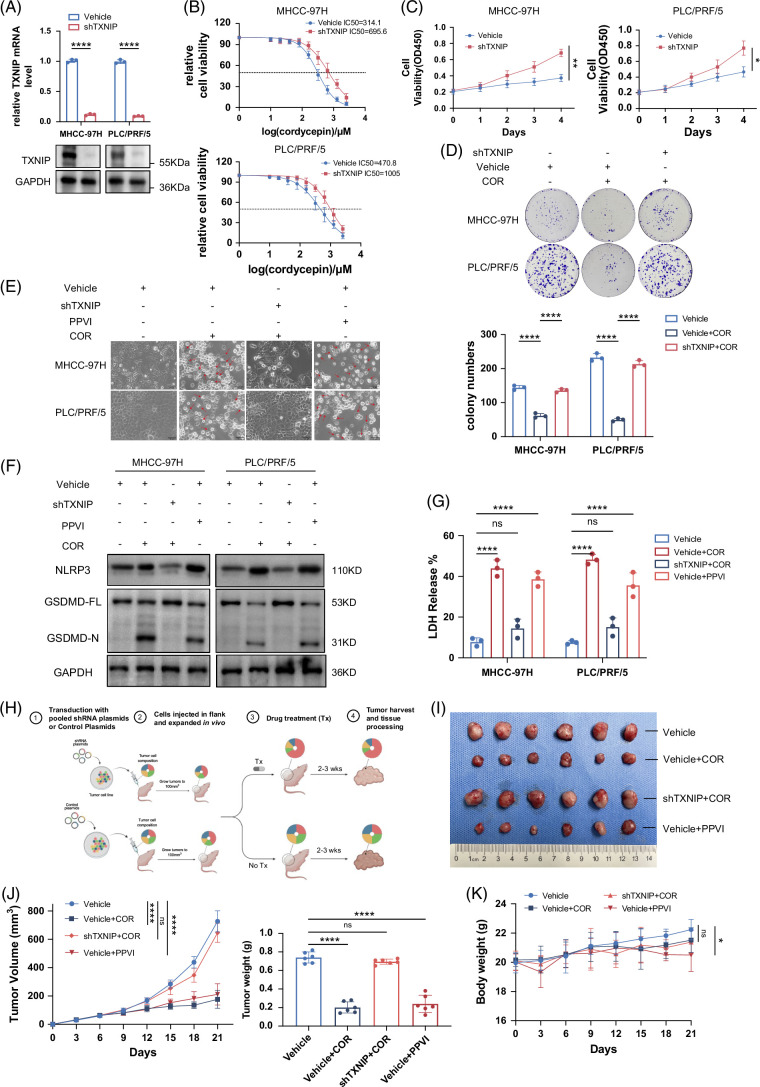
COR induces pyroptosis in HCC cells through TXNIP. (A) Western blot and qPCR showing the efficiency of TXNIP knockdown in MHCC97H PLC/PRF/5 cells. (B) IC50 of COR in control and TXNIP-knockdown cells. (C) Viability of TXNIP-knockdown cells compared with control cells after COR treatment. The COR concentration was 350 µM for MHCC97H cells and 500 µM for PLC/PRF/5 cells. (D) Effect of TXNIP knockdown on colony formation by PLC/PRF/5 and MHCC97H cells treated with COR. (E–G) Effects of TXNIP knockdown on cell pyroptosis, including pyroptotic morphology (E), GSDMD cleavage (F), and LDH release (G), were assessed in PLC/PRF/5 and MHCC97H cells treated with COR. (H) Schematic diagram of the PLC/PRF/5 subcutaneous xenograft model with TXNIP knockdown under COR treatment. The illustration was created with BioRender.com. (I) Knockdown of TXNIP enhances resistance to COR in the PLC/PRF/5 subcutaneous xenograft model. (J) Changes in tumor volume over time and differences in the weights of subcutaneous tumors among different groups. (K) Mouse body weights in the above subcutaneous xenograft model. The above data are presented as the means ± SDs of 3 independent experiments or triplicate experiments. *p* values were determined by a 2-tailed unpaired *t* test in (B, C), 1-way ANOVA in (D), or 2-way ANOVA in (G, J, and K), with significance denoted as follows: ns, not significant, *p* > 0.05; *p < 0.05; **p < 0.01; and ****p < 0.0001. Abbreviations: COR, cordycepin; LDH, lactate dehydrogenase; TXNIP, thioredoxin-interacting protein.

To confirm the crucial role of TXNIP in cellular pyroptosis, we knocked down TXNIP in MHCC97H and PLC/PRF/5 cells (Figure 3A). We subsequently compared the IC50 values of COR in TXNIP knockdown cells with those in control cells. The results indicated that TXNIP knockdown conferred resistance to COR in HCC cells (Figure 3B). TXNIP knockdown also restored the proliferative capacity of HCC cells under COR treatment (Figures 3C, D). In addition, TXNIP knockdown reduced the extent of HCC cell pyroptosis (including pyroptotic morphology [Figure 3E], GSDMD cleavage [Figure 3F], and LDH release [Figure 3G]) induced by COR. In vivo, tumors under COR treatment derived from TXNIP-knockdown cells were larger and grew faster than those derived from control cells (Figures 3H–J). Interestingly, COR exhibited a safety profile superior to that of the pyroptosis inducer PPVI (Figure 3K). These results suggest that COR induces pyroptosis in HCC cells through the upregulation of TXNIP.

### Low expression of TXNIP is related to a poor HCC prognosis

**FIGURE 4:**
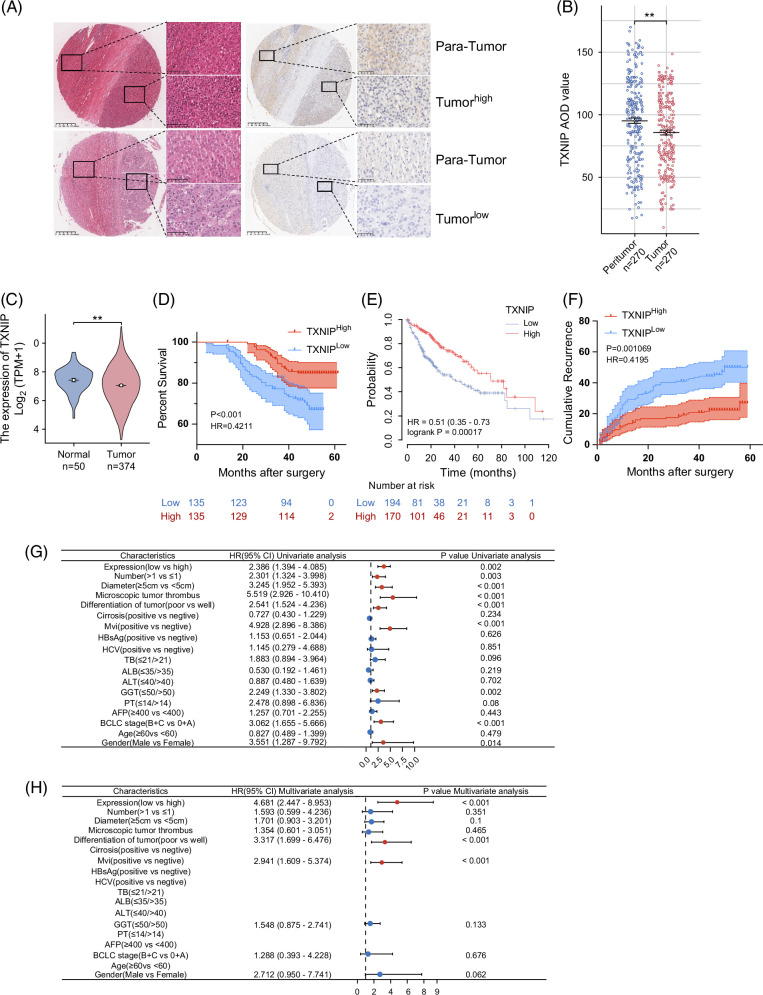
Low expression of TXNIP is related to a poor HCC prognosis. (A) Represent images of IHC staining with TXNIP-specific antibody and H&E staining in the TMA cohort. (B) Average OD values of the IHC images from our TMA. IHC images were analyzed using ImageJ. (C) Analysis of TXNIP expression from the TCGA database revealed a decreased level of TXNIP in HCC tumor tissue compared with normal tissue. (D) The relationship between overall survival and the expression of TXNIP was analyzed through Kaplan-Meier analysis. (E) Kaplan-Meier estimate of overall survival in the cohort with different levels of TXNIP from the prognostic data of the TCGA database. (F) Kaplan-Meier estimate of cumulative recurrence in the cohort stratified by different levels of TXNIP in the TMA cohort. (G, H) Univariate (G) and multivariate (H) Cox analyses of the OS of patients with HCC. *p* values were determined by a 2-tailed unpaired *t* test (B, C), and log-rank tests were performed (D–F). Significance is denoted as follows: ns, not significant, *p* > 0.05; **p < 0.01. Abbreviations: COR, cordycepin; IHC, immunohistochemistry; TMA, tissue microarray; TXNIP, thioredoxin-interacting protein.

Since TXNIP plays an important role in promoting pyroptosis in HCC cells, we wondered whether TXNIP expression is correlated with patient prognosis. We performed an immunohistochemistry analysis of our HCC tissue microarray and found that the overall expression of TXNIP in adjacent noncancerous tissues was significantly higher than that in paired cancerous tissues (Figures 4A, B). Data from the TCGA database also supported these results (Figure 4C). We subsequently used a dichotomous method to categorize the patients into a high TXNIP expression group and a low TXNIP expression group (Figure 4A). Kaplan-Meier analysis revealed that patients with high TXNIP expression had longer overall survival compared to those with low TXNIP expression (*p* < 0.001) (Figure 4D), a finding further supported by prognostic data from the TCGA database (Figure 4E). In addition, patients with low TXNIP expression levels tended to experience early recurrence after surgery (Figure 4F). Moreover, univariate analysis revealed that low TXNIP expression, tumor numbers >1, poor tumor differentiation, and microvascular invasion were risk factors for OS in patients with HCC (Figure 4G, red dots). In the multivariate Cox proportional hazards model, low TXNIP expression, poor tumor differentiation, and microvascular invasion were independent prognostic indicators of OS (Figure 4H). These results indicated that low TXNIP expression is associated with poor HCC prognosis.

### COR increases CD8^+^ T-cell infiltration, upregulates PD-L1 expression, and synergizes with anti–PD-L1 immunotherapy

Pyroptosis is a highly inflammatory mode of programmed cell death that can lead to the massive release of cellular contents and proinflammatory factors, which in turn activates a strong inflammatory response, stimulates other immune cells, and leads to tissue inflammation.^24^ Our previous experiments demonstrated that COR induces HCC pyroptosis through the upregulation of TXNIP. Therefore, we next investigated whether COR affects the tumor immune microenvironment. Using data from the TCGA database, we found that TXNIP expression was downregulated in multiple tumors compared to that in corresponding normal tissues (Figure 5A), suggesting that TXNIP can suppress cancer progression. Analysis of the TCGA database using the CIBERSORT method revealed a significant increase in CD8+ T-cell infiltration in the TXNIP high-expression group (Figure 5B), which was validated by 2 other databases (Figure 5C). Furthermore, positive correlations between TXNIP expression and CD8^+^ T-cell–associated chemokines CXCL9 and CXCL10 were observed (Figure 5D). Notably, compared with the expression of the other immune checkpoint molecules PD-1, TIGIT, and CTLA-4, the expression of TXNIP was more strongly positively correlated with the expression of PD-L1 in HCC tissues (Figure 5E). Based on the above findings, we hypothesized COR treatment could improve the antitumor efficacy of PD-L1 blockade. C57BL/6 mice bearing Hepa1-6 subcutaneous tumors or orthotopic tumors were treated with vehicle, atezolizumab (PD-L1 inhibitor), COR, or the combined treatment (Figure 5F). The results showed that the combination of atezolizumab and COR therapy further reduced tumor weight compared to monotherapy (Figures 5G, H, Supplemental Figures S3A, B, http://links.lww.com/HC9/B879). In addition, we assessed the safety of COR or anti–PD-L1 monotherapy and combination treatment in our HCC mouse model. Both single and combined treatments were well tolerated, with no significant body weight loss observed. (Figure 5I). Multiple immunofluorescence staining on subcutaneous and orthotopic tumors confirmed the promotion of CD8^+^ T-cell infiltration in the combination therapy compared with the monotherapy (Figures 5J, K, Supplemental Figures S3C, D, http://links.lww.com/HC9/B879). In addition, compared with the vehicle or atezolizumab, COR treatment increased the expression of PD-L1 in both subcutaneous and orthotopic tumors, which may be the potential mechanism by which COR synergizes with anti–PD-L1 immunotherapy (Figure 5L, Supplemental Figure S3E, http://links.lww.com/HC9/B879). However, this trend was not evident for PD-1. Moreover, we used an ELISA kit to detect the secretion of CXCL9 and CXCL10 in the subcutaneous and orthotopic tumor models. The results showed that the combination of atezolizumab and COR therapy further increased the secretion of CXCL9 and CXCL10, which partly explains why the combination therapy increased CD8^+^ T-cell infiltration (Figure 5M, Supplemental Figure S3F, http://links.lww.com/HC9/B879). Because GZMB^+^ CD8^+^ T cells play a role in the ability of infiltrating CD8^+^ T cells to kill tumors,^25^ we used flow cytometry to count GZMB+ CD8+ T cells in mouse tumors (Figure 5N). The results revealed that the percentage of GZMB+ CD8+ T cells among CD8+ T cells was significantly higher in the combined treatment group than in the other groups (Figure 5O). Based on this evidence, we demonstrated the efficacy and safety of combined COR and anti–PD-L1 treatment in preclinical HCC models.

FIGURE 5COR increases CD8^+^ T-cell infiltration, upregulates PD-L1 expression, and synergizes with anti–PD-L1 immunotherapy. (A) TXNIP mRNA expression in various cancer tissues in the TCGA database. (B) Infiltration of various immune cells with different TXNIP expression levels in the HCC database of TCGA. (C–E) Correlations between TXNIP expression and CD8+ cell infiltration (C), as well as with the expression of CXCL9, CXCL10 (D), PD-L﻿1, PD-1, TIGIT, and CTLA-4 (E). The data were analyzed with TIMER 2.0. (F–I) Schematic diagram (F), tumor images (G), tumor growth curves, tumor weights (H), and mouse body weights (I) of mice with Hep1–6 subcutaneous xenografts from the combination therapy experiments. Cordycepin was administered at 150 mg/kg through oral gavage, and atezolizumab was administered at 10 mg/kg through i.p. injection on the sixth day after Hep1–6 cell inoculation. The s﻿chematic diagram was created with BioRender.com. (J–L) Representative mIF images (J) and statistics (K, L) of CD8, PD-1, and PD-L1 expression in different therapy experiments. Each yellow dot represents 1 CD8^+^ T cell for the infiltration calculation in (K). (M) The expression levels of Cxcl9 and Cxcl10 in the previous tumors were determined by ELISA assays. (N, O) Flow cytometry analysis revealed a significant increase in the percentage of GZMB+ CD8+ T cells among the CD8+ T cells in the combined treatment group compared to the other groups. The data are presented as the means ± SDs; *p* values were determined by a 2-tailed unpaired *t* test in (A, B); Pearson correlation analysis was performed in (C–E). *p* values were determined by 1-way ANOVA in (H right, I, K, L, M, and O), or 2-way ANOVA in (H left), with significance denoted as follows: ns, not significant, *p* > 0.05; *p < 0.05; **p < 0.01; ***p < 0.001; ****p < 0.0001. Abbreviations: mIF, multiple immunofluorescence; TXNIP, thioredoxin-interacting protein.

**Figure:**
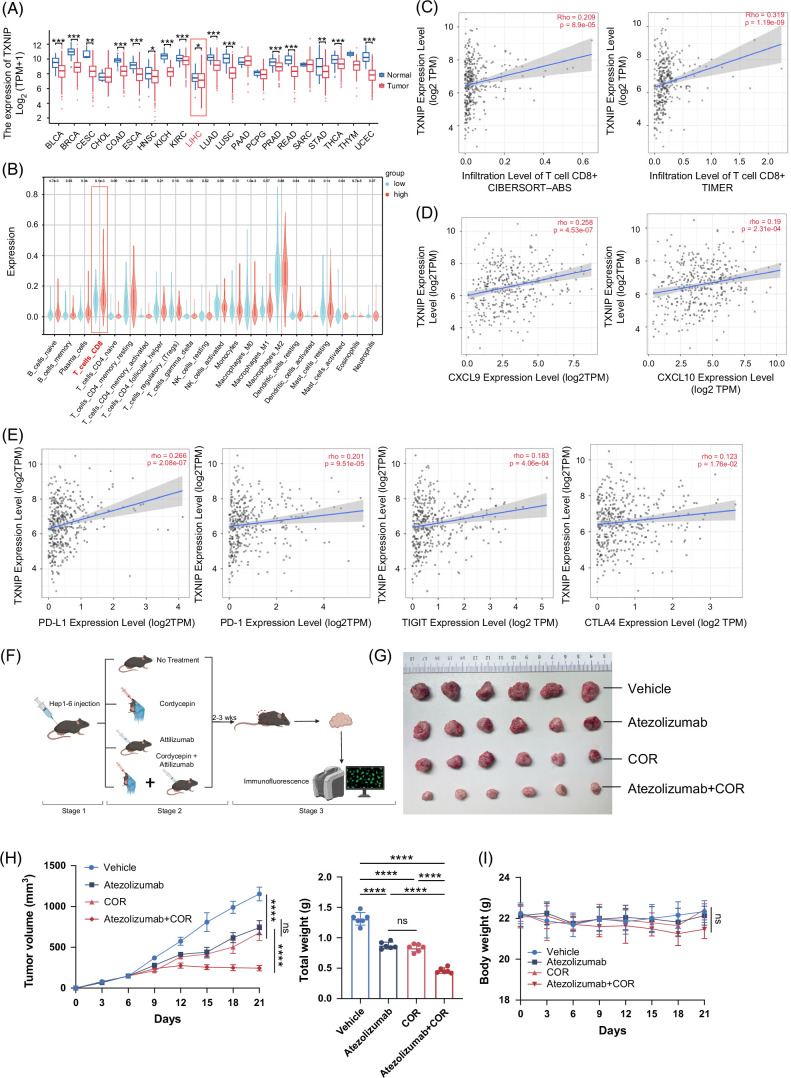

**Figure:**
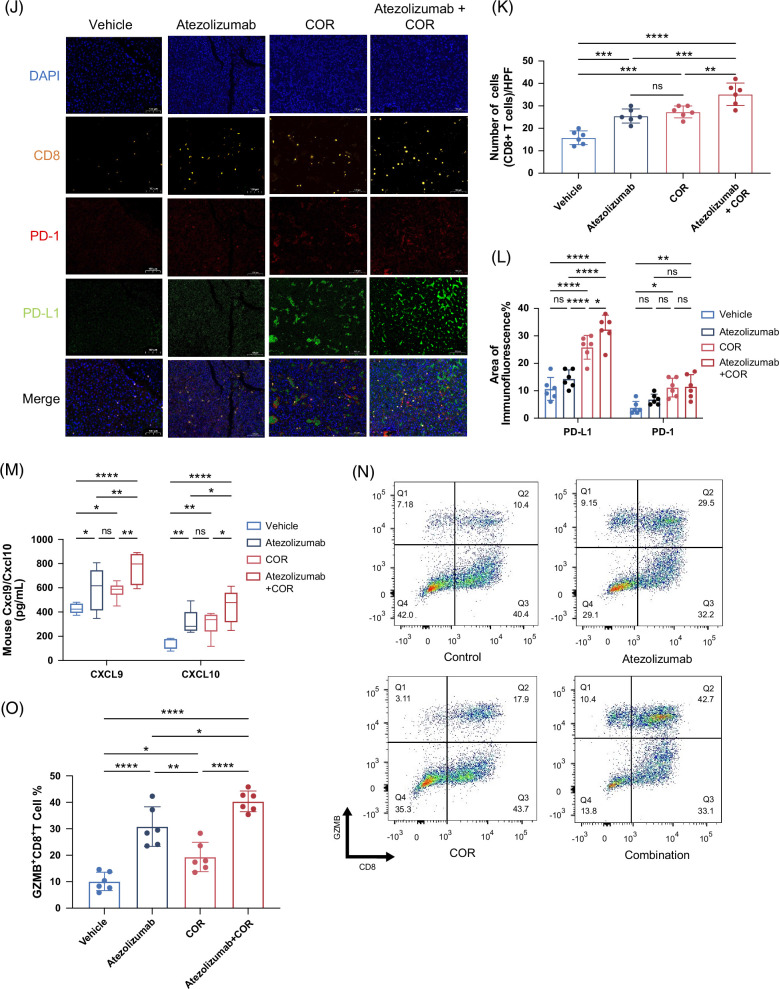

## DISCUSSION

**FIGURE 6:**
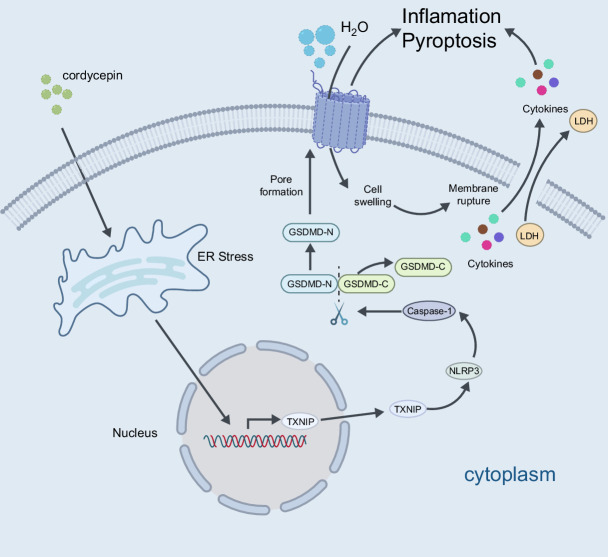
Schematic diagram of the molecular mechanism by which COR induces pyroptotic cell death through the activation of the TXNIP/NLRP3/GSDMD signaling axis in HCC cells. The s﻿chematic diagram was created with BioRender.com. ﻿Abbreviation: COR, cordycepin.

COR, a natural product of *Cordyceps militaris*, has been extensively studied and shown to have potential antitumor activity since its discovery by German scientists.^6^ Recent studies have shown that COR may affect the progression of tumor cells through many pathways, including cell cycle arrest, apoptosis induction, and DNA damage.^7^,^8^,^9^ The effect of COR on the tumor cell cycle was also confirmed by our RNA sequencing results (Figure 2B). In addition to its antitumor effects, COR can ameliorate NASH^26^ and inhibit cell senescence^27^, further enhancing the potential application value of COR. However, to date, the mechanism by which COR induces tumor cell pyroptosis has not been explored, and the mechanism underlying this process has not been elucidated. Here, we first proposed and confirmed that COR can induce pyroptosis in HCC cells. The possible underlying mechanisms were explored (Figure 6).

We performed RNA sequencing of COR-treated HCC cells to investigate the mechanism by which COR induces tumor cell death and determined that ERS and expression of TXNIP was significantly upregulated in cells cultured with COR. ERS is a cellular condition that arises when misfolded or unfolded proteins accumulate within the endoplasmic reticulum and can be triggered by various exogenous or endogenous factors.^14^ TXNIP, also known as vitamin D3 upregulated protein 1 or thioredoxin-binding protein 2, was initially identified as a binding partner of thioredoxin and could be upregulated under various stress condition^15^,^28^. Several studies have documented that TXNIP serves as a key link between cellular stress and inflammatory responses and is also a key pathological regulator of several diseases, including diabetes and neurodegenerative diseases.^15^,^22^,^28^ Here, we observed that TXNIP can mediate pyroptosis and confirmed the positive correlation between TXNIP expression and favorable prognosis in patients with HCC using clinical data, indicating that TXNIP may be a potential therapeutic target in HCC. However, the mechanisms by which COR causes high TXNIP expression remain unexplored, providing a direction for future research.

COR is also known as 3′-deoxyadenosine, and we explored the effects of 3 COR analogs (adenosine, 2′-deoxyadenosine, and ddAdo) on HCC cells. Adenosine is a ubiquitous endogenous autocrine hormone and metabolite that acts through 4 G protein–coupled receptors, affecting virtually all aspects of cellular physiology, including neuronal activity, vascular function, platelet aggregation, and regulation of blood cells.^29^,^30^ Exogenous adenosine has rarely been reported to exert direct antitumor effects. 2′-Deoxyadenosine is a nucleoside derivative of adenosine,^31^ and ddAdo is an inhibitor of HIV replication.^32^ However, limited direct research has been conducted on their properties and effects on cancer cells. We confirmed that exogenous adenosine and 2′-deoxyadenosine had little inhibitory effect on HCC cells. However, surprisingly, ddAdo exerted significant antitumor effects, though these effects were not as strong as those of COR. This result suggests that the deletion of the hydroxyl group at the 3′ position is crucial for increasing adenosine toxicity. However, the deletion of the 2′-position hydroxyl group attenuates this effect.

Pyroptosis is a newly discovered form of inflammatory cell death, characterized by the cleavage of gasdermin family members into N-terminal fragments. These cleaved N-terminal fragments translocate and penetrate the cell membrane, leading to cell swelling, cell death, and release of inflammatory factors^13^,^33^. This process typically relies on the activation of the NLRP3 inflammasome complex. The NLRP3 inflammasome converts pro-Caspase-1 into its active form (cleaved Caspase-1), which in turn cleaves pro-IL-1β and pro-IL-18 and GSDMD.^13^ Several drugs, including PPVI,^23^ punicalin,^34^ and ICy-Q,^35^ have been shown to promote pyroptosis. Here, we propose the use of COR, which is capable of inducing pyroptosis. However, its analog, ddAdo, did not perform as well in this regard. Our findings suggest that COR may have a unique structure that allows it to effectively activate the pyroptosis pathway in HCC cells. In contrast, the lack of this property in ddAdo may lead to its limited role in activating pyroptosis mechanisms. These findings also provide important clues to improve our understanding of the different potential activities of these compounds in antitumor therapy.

Immunotherapy has emerged as a frontline pharmacological approach for HCC treatment. PD-L1 plays a pivotal role in immunomodulation. Its primary function is to regulate immune response by binding to its receptor PD-1 (programmed cell death protein 1). When PD-L1 binds to PD-1, it suppresses T-cell activation, proliferation, and cytotoxicity, leading to immune tolerance and evasion, which are critical mechanisms in tumor immune escape.^36^ Drugs that disrupt the interaction between PD-L1 and PD-1, such as anti–PD-L1 and anti–PD-1 antibodies, have gained widespread clinical use and have shown remarkable therapeutic efficacy. These agents work by reinstating T-cell immune surveillance against tumors, thereby promoting the immune cell–mediated killing of tumor cells and ultimately achieving disease control.^18^ However, although PD-1/PD-L1 inhibitors have achieved significant therapeutic benefits for some patients, many continue to develop drug resistance after treatment.^19^ Solutions for overcoming anti–PD-L1 resistance represent a critical focus of ongoing immunotherapy research. Our study revealed that COR can synergize with anti–PD-L1 immunotherapy by increasing CD8^+^ T-cell infiltration and upregulating PD-L1 expression. These changes may be due to cellular pyroptosis induced by COR, which is an inflammatory response that leads to the release of inflammatory factors.^37^ However, we could not elucidate why COR leads to high PD-L1 expression. This process may also be a direction for future research. The combination of anti–PD-L1 and COR yielded better therapeutic outcomes than monotherapy. This new finding provides useful insights into the future development of tumor immunotherapy.

## CONCLUSIONS

In summary, our study confirmed the inhibitory effect of COR on HCC, and, for the first time, we showed that COR achieved this effect by increasing TXNIP-induced tumor cell pyroptosis. In addition, by increasing the infiltration of T cells and PD-L1 expression in HCC, COR can synergize with anti–PD-L1 immunotherapy. Therefore, the combination of anti–PD-L1 and COR therapies may benefit patients with HCC.

## Supplementary Material

## Data Availability

The data will be made available upon request. Sequencing data supporting the findings of this study have been deposited in the Sequence Read Archive (SRA) with the primary accession number SRP517566. The processed sequencing data are displayed in the Supplemental Table, http://links.lww.com/HC9/B880. The publication and licensing rights for the figures created with BioRender.com are provided in the Supplementary Information, http://links.lww.com/HC9/B879. Ai-Wu Ke and Wen-Zheng Qin designed and supervised the experiments. Chang-Jun Tan, Guo-Huan Yang, and Wen-Xin Xu collected clinical data. Bu-Gang Liang, Hong-Ye Shen, and Yi-Ming Zheng performed the experiments and analyzed the data. Bu-Gang Liang wrote the manuscript All the authors have read and approved the final manuscript. This study was supported by the Shanghai Municipal Natural Science Foundation (22ZR1411200) and the National Natural Science Foundation of China (grant no. 82172799). The authors have no conflicts to report. All research was conducted in accordance with the Declaration of Helsinki and the Declaration of Istanbul. All studies were approved by the Zhongshan Hospital Research Ethics Committee. Written informed consent was obtained from all patients.
