# Supplementary material for: Cordycepin mediates pyroptosis in HCC through the upregulation of TXNIP and synergizes with anti–PD-L1 immunotherapy
Source: Hepatol Commun. 2025 Feb 26;9(3):e0633. doi: 10.1097/HC9.0000000000000633 (PMC11868431; doi:10.1097/HC9.0000000000000633)
Supplement: Supplementary file 1 [file hc9-9-e0633-s001.docx]

Supplemental information

Manuscript Number: HEP4-24-0745.R2

**methods**

Cell lines and transfection

The PLC/PRF/5 cell line from the American Typical Cultures Conservation Center (ATCC, Manassas, VA). The MHCC97H cell line and mouse HCC cell line Hepa16 were obtained from the Cell Bank of the Chinese Academy of Sciences. All cell lines were cultured in supplier-recommended media supplemented with 10% fetal bovine serum (Gibco, South American origin), 100 μg/mL penicillin/streptomycin (Yeasen, Shanghai, China), at 37°C in a humid environment containing 5% CO_2_. For shRNA transfection, cells were plated at 60%-80% confluence in Gibco serum-free medium and virus was added for transfection. Cell lines were selected in the presence of 5 μg/ml puromycin. Forty-eight hours after transfection, the cells were used for functional assays. shTXNIP and non-target shRNA control cloned in a lentiviral vector were purchased from Sigma (St. Louis, USA). The sequence of human shTXNIP is 5'- CCGACTTATACTGAGGTGGAT -3'.

Acquisition of drugs

Cordycepin, Polyphyllin VI (PPVI), Atezolizumab, adenosine, 2'-deoxyadenosine, and 2',3'-dideoxyadenosine (ddAdo) was purchased from MCE (HY-N0262, HY-N0816, HY-P9904, HY-B0228, HY-W040329, HY-W013441, America). The drug is dissolved in the medium according to the instructions from suppliers.

Elisa

Tumors were ground into suspensions and after centrifuging the supernatant was collected for ELISA detection. Mouse CXCL9 ELISA Kit (ab203364, abcam, America) and Mouse IP-10 ELISA Kit (CXCL10) (ab260067, abcam, America) were used according to the manufacturer’s protocol.

Flow cytometry

The tumors obtained from the mouse model were minced and digested with collagenase and trypsin to obtain a single-cell suspension. The cells were then stained with a fixable viability dye and permeabilized, followed by incubation with fluorochrome-conjugated antibodies in the dark for 30 minutes. The cells were subsequently analyzed using a BD FAC-SAria III flow cytometer. The results were analyzed using FlowJo software. The following antibodies were used: anti-Granzyme B antibody (EPR22645-244, abcam, America), anti-CD8 alpha antibody (RM1129, abcam, America).

RNA extraction and qPCR

Total RNA was extracted from HCC cells using TRIzol reagent (Sigma-Aldrich, USA) according to the manufacturer's protocol. cDNA was synthesized using random primers and the HifairII First Strand cDNA Synthesis Kit (Yeasen, Shanghai, China). Real-time fluorescence polymerase chain reaction (qPCR) was performed using HieffqPCR SYBR green master mix (Yeasen, Shanghai, China). The primer sequences used were GAPDH forward 5'-ATGTTCGTCATGGGTGTGAA-3', antisense 5'-GGTGCTAAGCAGTTGGGTGGGT-3'. TXNIP forward 5'- ACGGGTAATAGTGGAAGT -3', antisense 5'- CCCAGTAGTCTACGCAAC -3'.

Protein extraction and Western blotting.

Cells or tissue samples were harvested and transferred to centrifuge tubes. Add lysis buffer (Beyotime Institute of Biotechnology, China) supplemented with protease inhibitors (Beyotime Institute of Biotechnology, China) to the samples. Protein concentrations were determined using the BCA protein quantification kit (Yeasen, Shanghai, China). Proteins were separated by SDS-PAGE and transferred to 4.5 µm aperture polyvinylidene difluoride (PVDF) membranes (Millipore, USA). The membranes were incubated overnight at 4°C with primary antibodies. Next day, the membrane was incubated with the corresponding secondary antibody for 1 h at room temperature. The protein bands were detected using a chemiluminescent substrate and were visualized using an imaging system. The following antibodies were used: anti-TXNIP antibody (1:2000, abcam, EPR14774), anti-NLRP3 antibody (1:1000, abcam, EPR23094-1), anti-GSDMD (1:1000, cst, E8G3F), anti-GAPDH antibody (1:1000, cst, D16H11), anti-Rabbit secondary antibody (1:5000, proteintech, RGAR001), anti-Mouse secondary antibody (1:5000, proteintech, RGAM001).

Colony formation assays, Cell Counting Kit-8(cck8) assays and IC50.

For colony formation assays, an initial seeding density of 300 cells was plated into individual wells of 24-well plates (or 2000 cells in 6-well plates) and cultured in 10% FBS-supplemented Dulbecco's modified Eagle's medium (DMEM) or dosed medium for a duration of 12 days. Following incubation, colonies were fixed with paraformaldehyde and subsequently stained with a solution containing 0.1% crystal violet for precisely 15 minutes. Clones demonstrating the presence of more than 30 cells were deemed as positive, indicative of robust colony formation. To assess cell viability, the Cell Counting Kit-8 (DOJINDO, Shanghai, China) was utilized. Cells were cultured in a 96-well microplate at a density of 1000 cells per well and allowed to adhere overnight. Subsequently, cells were treated with different concentrations of adenosine, 2'-deoxyadenosine, cordycepin and 2',3'-dideoxyadenosine for 24, 48, 72, and 96 hours. Following the respective treatment durations, CCK8 reagent was added to each well and incubated for 2 hours at 37°C and absorbance at 450nm was measured using a microplate reader. For IC50 assays, 1000 cells were seeded into individual wells of a 96-well plate and allowed to adhere. Subsequently, cells were treated with varying concentrations of drugs (0, 10, 20, 40, 80, 160, 320, 640, 1280, 2560 and 5120μM) for 48 hours at 37°C. Then, cells were stained by incubating with a mixture of CCK-8 and DMEM solution (10 μl CCK-8 in 100 μL DMEM). Absorbance at 450nm was then measured using a microplate reader. The half-maximal inhibitory concentration (IC50) value was determined by generating a cell growth curve.

Wound healing migration and transwell invasion assays

For wound healing migration, Cells were seeded into 6-well plates and allowed to grow until reaching approximately 90% confluence. Using a sterile pipette tip, made a scratch across the monolayer of cells. The cells were then washed with phosphate-buffered saline (PBS) to remove any detached cells and subsequently cultured in DMEM supplemented with 1% fetal bovine serum (FBS) and different drugs. Cell migration was monitored using an inverted microscope (Olympus, Tokyo, Japan), with images captured immediately (0 hours) and 48 hours post-scratch. For invasion assays, 24-well plates with inserts containing 8 μm pores (Corning, New York, USA) were used. The upper chamber was pre-coated with 50μL of extracellular matrix gel. After allowing the gel to solidify for 30 minutes, 5000 cells suspended in 200 μL of serum-free medium were seeded into the upper chamber, while the lower chamber was filled with 500 μL of medium supplemented with 10% fetal bovine serum (FBS). The plates were then incubated at 37°C in a humidified atmosphere with 5% CO_2_ for 72 hours. After incubation, the cells that invaded through the membrane were fixed with 4% paraformaldehyde and stained with 0.1% crystal violet for 30 minutes. The invaded cells were visualized and counted under an optical microscope (Olympus, Tokyo, Japan).

Multiple immunofluorescence staining(mIF)

For mIF, the tumor slide was first deparaffinized using dimethylbenzene and graded alcohols (95%, 85%, 75%), and rinsed with PBS three times. It was then incubated with 0.3% hydrogen peroxide and then subjected to antigen retrieval using citrate buffer at 95°C for 15 minutes, followed by blocking with 5% BSA. Primary antibodies were incubated at 37°C in a humidified chamber for 60 minutes, and then incubated with corresponding TSA fluorescent dye was applied to the sample area. The process was repeated with different primary antibodies and fluorescent dyes for multiplex immunofluorescence. Finally, the slide was incubated with DAPI solution at 37°C in darkness for 10 minutes. CaseViewer software was used to detect and capture images. The following antibodies were used：Anti-CD8 alpha(1:1000, abcam, EPR21769), anti-PDL1(1:200, abcam, EPR20529), anti-PD1(1:250, abcam, RM1129)

RNA sequencing

MHCC-97H cells were cultured in the normal or drug-treated medium for two days, respectively. Subsequently, samples from both control and experimental groups were sent to Personalbio company in China for sequencing. Three replicates were prepared for each group. The sequencing results were then analyzed using the R language.

**Supplementary Figure S1**


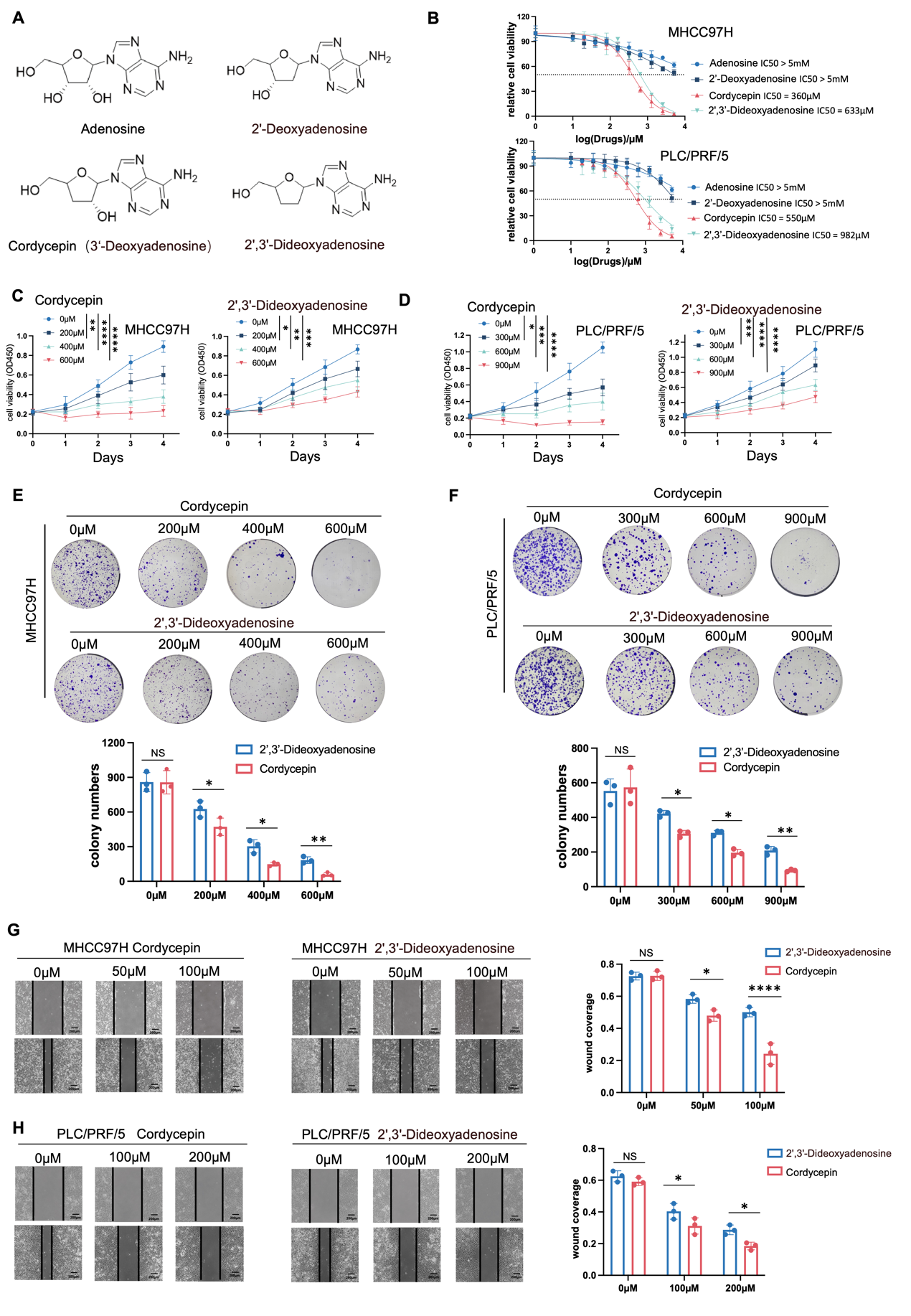


**Supplementary Figure S1. Effects of COR analogs on HCC cells compared to COR**

(A) The chemical formula of adenosine, 2'-deoxyadenosine, 3'-deoxyadenosine (COR) and 2',3'-dideoxyadenosine (ddAdo). (B) Determination of IC50 of adenosine, 2'-deoxyadenosine, 3'-deoxyadenosine and 2',3'-dideoxyadenosine on MHCC97H and PLC/PRF/5. (C-D) Cell viability after treatment with various concentrations of 3'-deoxyadenosine or 2',3'-dideoxyadenosine determined by CCK-8 assay of MHCC97 and PLC/PRF/5 cells. (E-F) The colony formation assay results of MHCC97 and PLC/PRF/5 cells treated with various concentrations of 3'-deoxyadenosine or 2',3'-dideoxyadenosine. (G-H) Scratching assay for cell migration of MHCC97 and PLC/PRF/5 cells treated with various concentrations of 3'-deoxyadenosine or 2',3'-dideoxyadenosine.

**Supplementary Figure S2**


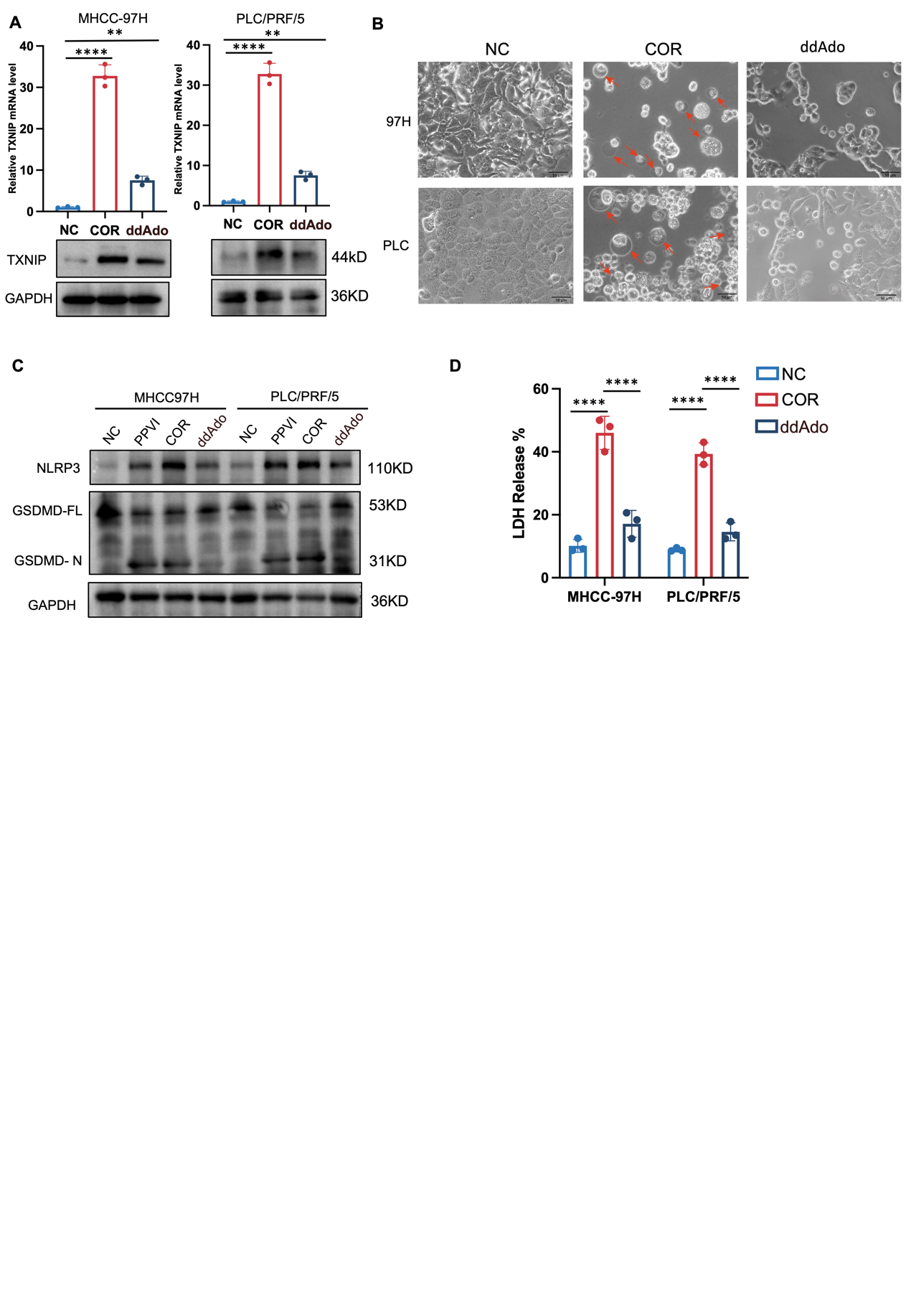


**Supplementary Figure S2. Induction of cell pyroptosis by 2',3'-dideoxyadenosine or cordycepin**

(A) TXNIP expression after treatment with half-maximal inhibitory concentrations of COR or ddAdo in MHCC97 and PLC/PRF/5 cells. (B-D) MHCC97H and PLC/PRF/5 cell lines were pretreated with Vehicle, or COR (350µM for MHCC97H and 500µM for PLC/PRF/5), or ddAdo (600µM for MHCC97H and 1000µM for PLC/PRF/5). Pyroptosis was detected with morphology (B, red arrows indicate pyroptosis cells), GSDMD cleavage (C), and LDH release (D).

**Supplementary Figure S3**


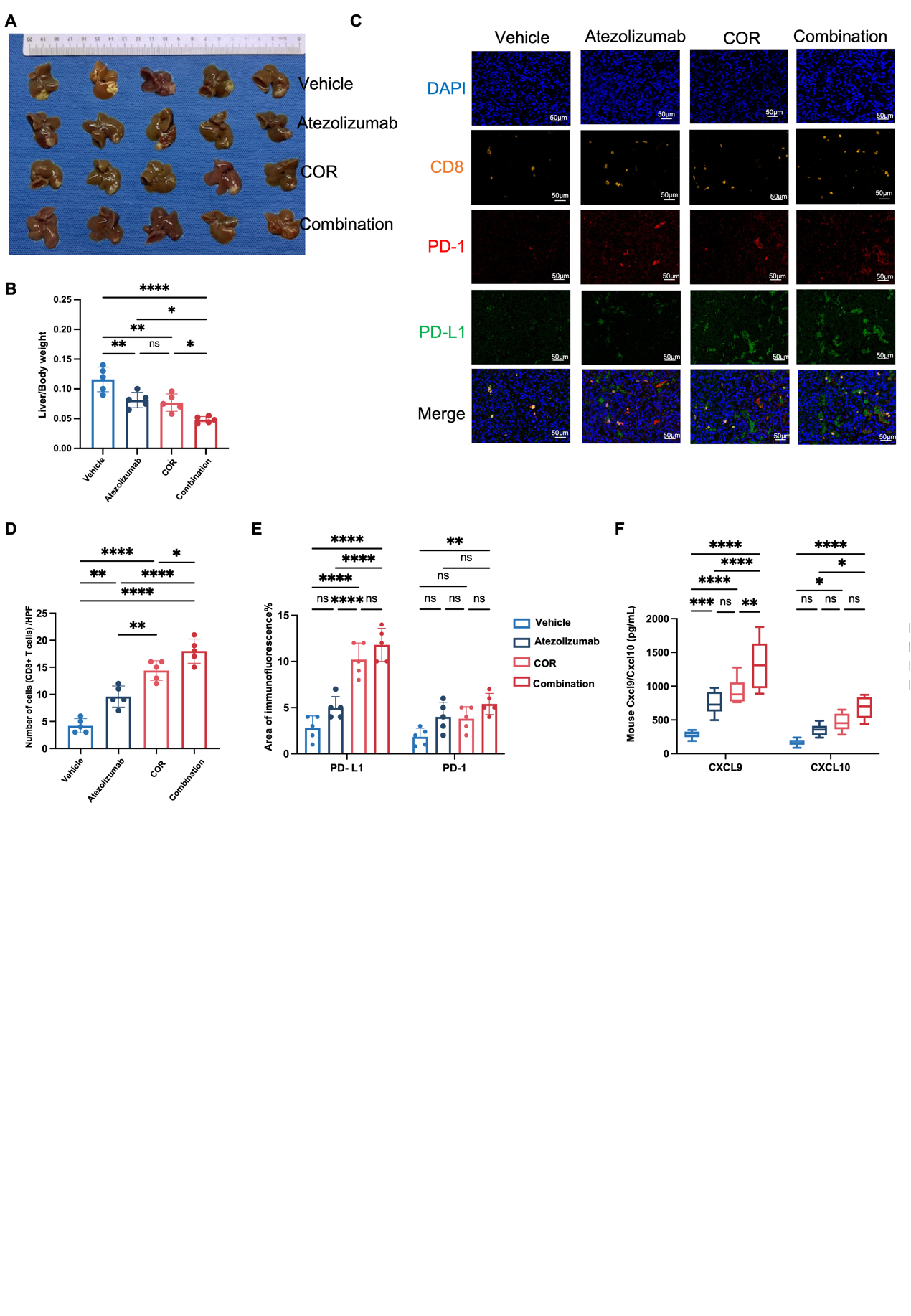


**Supplementary Figure S3. COR increases CD8^+^ T-cell infiltration, upregulates PD-L1 expression and synergizes with anti-PD-L1 immunotherapy in orthotopic HCC models**

(A-B) Image (A) and liver/body weight (%) (B) of orthotopic Hepa1-6 tumor models treated with vehicle or atezolizumab or COR or the combination of atezolizumab and COR (n = 6 per group). (C-E) Representative mIF images (C) and statistics (D and E) of CD8, PD-1 and PD-L1 expression from the previous orthotopic Hepa1-6 tumor models in different therapy groups. (F) The expression levels of CXCL9 and CXCL10 in the previously treated tumors determined by ELISA assays.

Publication licenses
